# Supplementary material for: Differential Expression of Genes Related to Growth and Aflatoxin Synthesis in Aspergillus flavus When Inhibited by Bacillus velezensis Strain B2
Source: Foods. 2022 Nov 13;11(22):3620. doi: 10.3390/foods11223620 (PMC9689179; doi:10.3390/foods11223620)
Supplement: Supplementary file 1 [file foods-11-03620-s001.zip › foods-1976175-supplementary.pdf]

Supplementary figures

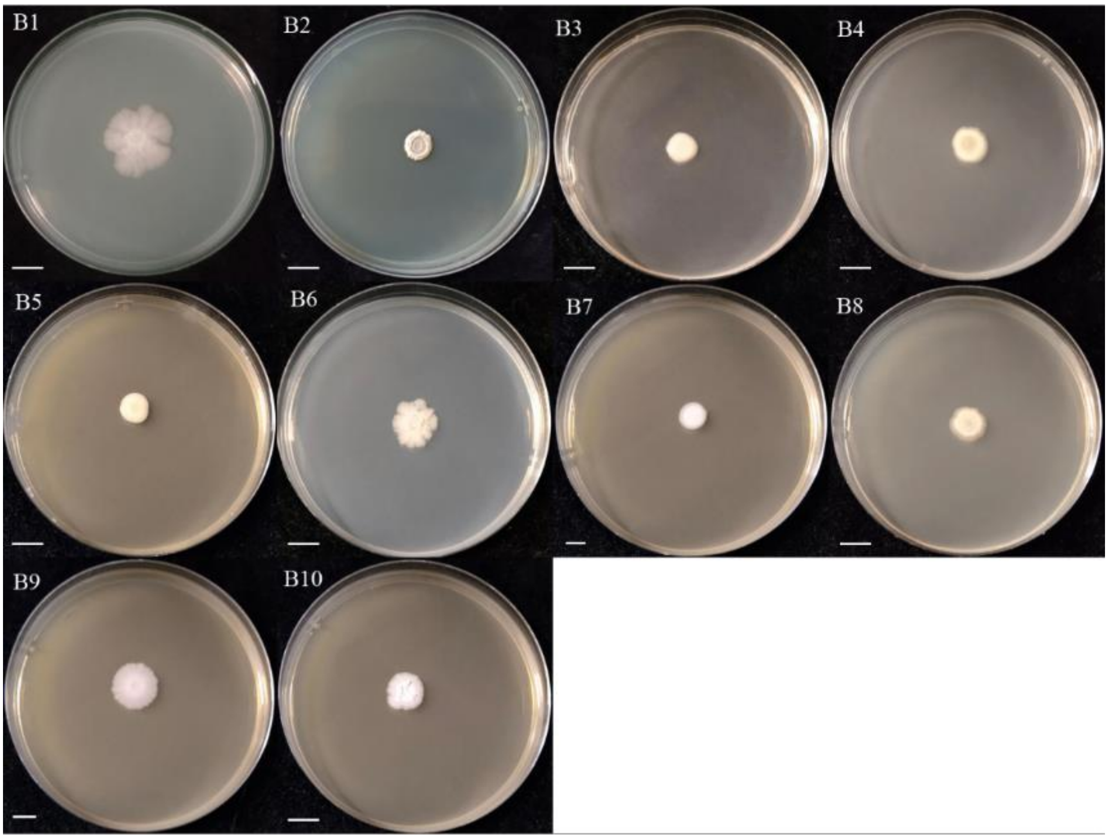

Figure S1: Morphology of colonies of bacterial isolates obtained from the *Camellia sinensis*. Colony pictures were taken at 4 days post-inoculation by applying 5 μL of a bacterial suspension on the center of a LB plate (diameter 9 cm). Scale bars represent 10 mm.

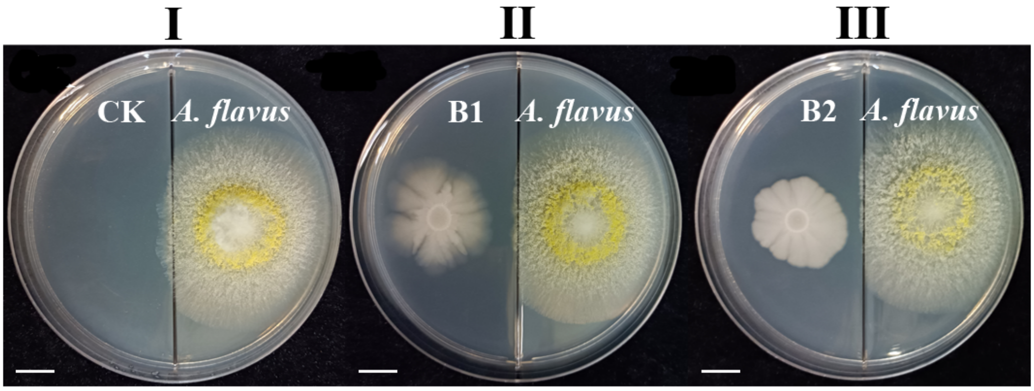

Figure S2: Inhibition effect of volatile compounds produced by B1 and B2 on the growth of *A. flavus*. Colony pictures were taken at 4 days post-inoculation by applying 5 μL of bacterial and *A. flavus* conidial suspension on the two sides of a LB plate (plate separated by a baffle), respectively. Scale bars represent 10 mm.

Table S1 Relative inhibition rates of *A. flavus* inhibited by bacterial isolates B1~B10. Datas statistics were performed at 4 days post-inoculation. Values appended by asterisks are significantly different (Student's *t* test: $P < 0.05$). All assays were repeated three times with three replicates per repeat.

Strain	Relative inhibition rate (%)
B1	34.22±0.0482*
B2	35.72±0.0060*
B3	0
B4	0
B5	0
B6	0
B7	0
B8	0
B9	0
B10	0

Table S2 Expression profiling genes involved in aflatoxin biosynthesis in *A.flavus*.

Query Gene ID	Gene ID	Gene Description	Log2FC
Unigene2277	*AFLA_033290*	Regulator of secondary metabolism LaeA	-0.13
Unigene19513	*AFLA_139370*	*aflB* / *fas-1* /fatty acid synthase beta subunit	-0.51
Unigene19011	*AFLA_139410*	*aflC* / *pksA* / *pksL1* / polyketide synthase	-0.78
Unigene4845	*AFLA_139390*	*aflD* / *nor-1*/ reductase	-4.12
Unigene11479	*AFLA_139310*	*aflE* / *norA* / *aad* /*adh-2* / NORreductase/dehydrogenase	0.46
Unigene7433	*AFLA_139260*	*aflG* / *avnA* / *ord-1*/ cytochromeP450 monooxygenase	-0.03
Unigene21431	*AFLA_139330*	*aflH* / *adhA* / short chainal cohol dehydrogenase	0.57
Unigene780	*AFLA_139320*	*aflJ* / *estA* / esterase	-2.13
Unigene13467	*AFLA_139190*	*aflK* / *vbs* / VERB synthase	-1.10
Unigene15485	*AFLA_139300*	*aflM* / *ver-1* / dehydrogenase/ketoreductase	-0.22
Unigene3205	*AFLA_139280*	*aflN* / verA / monooxygenase	0.26
Unigene4604	*AFLA_139220*	*aflO* / *omtB* / *dmtA* / O-methyltransferase B	-0.19
Unigene11091	*AFLA_139210*	*aflP* / *omtA* / *omt-1* /O-methyltransferase A	-0.79
Unigene19606	*AFLA_139200*	*aflQ* / ordA / *ord-1* / oxidoreductase / cytochromeP450 monooxigenase	-0.38
Unigene14983	*AFLA_139360*	*aflR* / *apa-2* / *afl-2* / transcription activator	-0.32
Unigene16697	*AFLA_139420*	*aflT* / transmembrane protein	-0.60
